# Dietary copper-driven colonic dysbiosis mediates oxidative stress and butyrate deficiency to facilitate the spread of resistome in pigs

**DOI:** 10.1038/s41522-026-00949-1

**Published:** 2026-03-05

**Authors:** Yang Wen, Meng Gao, Zhenyu Wang, Xiaoyi Liu, Yunhui Zhang, Gang Lin, Pingli He, Hua Yang, Yingping Xiao, Wentao Lyu

**Affiliations:** 1 State Key Laboratory for the Quality and Safety of Agro-Products, Zhejiang Provincial Key Laboratory of Agricultural Microbiomics, Institute of Agro-Product Safety and Nutrition, Zhejiang Academy of Agricultural Sciences, Hangzhou, China; 2 School of Life Sciences, Westlake University, Hangzhou, China; 3 State Key Laboratory of Animal Nutrition, Frontiers Science Center for Molecular Design Breeding (MOE), China Agricultural University, Beijing, China; 4 College of Environmental Science and Engineering, Tongji University, Shanghai, China; 5 Institute of Quality Standards and Testing Technology for Agricultural Products, Chinese Academy of Agricultural Sciences, Key Laboratory of Agrifood Safety and Quality, Ministry of Agriculture and Rural Affairs, Beijing, China

**Keywords:** Microbiology, Molecular biology

## Abstract

Copper-induced transmission of antimicrobial resistance has been well documented in livestock farming environments, but the in vivo mechanisms driving fecal resistome development remain unclear. Here, 120 mg/kg CuSO_4_ and copper-peptide were supplemented to piglets, and the fecal resistome development was first analyzed by metagenomic sequencing. In this study, dietary CuSO_4_ drove abundant and diverse ARGs and MRGs. Following CuSO_4_ deprivation, ARGs and copper resistance exhibited a persistent promotion, whereas most MRGs rapidly declined. The resistance development was characterized by abundant MGEs. This phenomenon expanded the multiple-antibiotic resistance reservoir in fecal community, which was preferentially harbored by pathogens. Furthermore, dietary CuSO_4_ disturbed colonic homeostasis, characterized by impaired epithelial integrity and reduced butyrate-producing bacteria abundance, which coincided with an oxidative stress environment and increased prevalence of multiple-resistant pathogens, such as *Escherichia coli* and *Enterococcus spp*. In vitro validation further supported these associations, showing that butyrate supplementation and hypoxic conditions alleviated Cu^2+^-induced ROS generation and reduced the frequency of ARGs conjugative transfer. Overall, this study suggests that dietary inorganic copper may contribute to microbial disturbances linked to oxidative stress and potentially facilitate antimicrobial resistance transmission among pathogens, highlighting organic copper as a sustainable alternative for mitigating resistance risks in farmed animals.

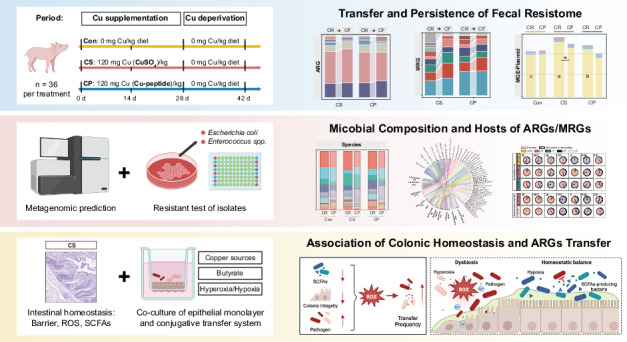

## Introduction

Based on the “one health” concept, the emergence and dissemination of antimicrobial resistance (AMR) in livestock production is now recognized as a critical public health challenge^1–3^. With the ban on antibiotics as growth promoters, some alternatives used in large quantities may still influence AMR by acting as co-selection drivers. Copper with broad-spectrum antimicrobial and growth-promoting properties is widely used in weaned piglets at levels exceeding basic metabolic requirements, relieving weaning stress^4–6^. Since little copper (about 10%) was absorbed in the intestine, residual copper might not only be excreted into agricultural ecosystems performing persistent selective pressures but also exert co-selection of ARGs and MRGs in the fecal microbiome^7^, contributing to compound resistance development. Addressing these issues requires a re-evaluation of dietary copper supplementation strategies in livestock production systems.

Antimicrobial resistance arises through the selection of naturally occurring resistant mutants and horizontal gene transfer^8^. While environmental stress, like heavy metals, is recognized drivers of this process, the effects are context-dependent. For example, studies on sub-lethal Cu^2+^ (20 mg/L) exposure suggested that copper-induced oxidative stress could activate the plasmid-mediated transmission of bacterial resistance^9,10^. Additionally, fecal resistome analyses in livestock environments indicate that mobile genetic elements (MGEs) could drive the co-transfer of antibiotic resistance within complex microbiomes in the presence of high levels of heavy metals^11,12^. Notably, co-occurrence of ARGs and MRGs is frequently observed in pathogens such as *E. coli* and *Enterococcus spp*^13^. In our previous study in pigs, dietary supplementation with 120 mg/kg CuSO_4_ was demonstrated to induce colonic barrier injury and enrich pathogenic bacteria^14^. This indicates that such intestinal conditions may provide the prerequisites for the ARGs transmission, such as pathogenic bacterial carriers and oxidative stress factors. This may expand our knowledge of trace minerals applied in the pig industry.

Although the addition of copper to feed has been restricted to approximately 120 mg/kg in the period of piglet, effective strategies to reduce the risks associated with excessive antimicrobial resistance are necessary. As a chelated form of copper bound to organic ligands, organic copper exhibits higher bioavailability, reduced environmental excretion, and potentially lower selective pressure on resistant bacteria^15^. Excitedly, addition of proteinate copper at 20 to 160 mg/kg could achieve similar or even stronger effects in promoting growth and reducing diarrhea than the same dose of tribasic copper chloride in pigs^16,17^. Despite these promising findings, the impact of organic copper on the fecal resistome and gut microbial ecology in swine remains underexplored, particularly in comparison to traditional inorganic copper sources.

This study aims to provide a comprehensive assessment of the prevalence and persistence of the fecal resistome in pigs under different copper supplementation strategies. By integrating analyses of resistome dynamics, microbial community composition, and intestinal homeostasis in piglets, this research seeks to elucidate the risk and its underlying mechanisms of inorganic copper-induced compound resistance pollution, and the role of organic copper as a sustainable strategy for balancing animal productivity with environmental and public health considerations.

## Methods

### Animals and sample collection

This study was conducted following the principles and guidelines for laboratory animal use from China Agricultural University. All animal experiments were approved by the Institutional Animal Care and Use Committee of the China Agricultural University, Beijing, China, under permit no. AW03002202-1-3 (Beijing, China). The weaned piglet experiment was carried out at the swine research unit of China Agricultural University in Hebei province. Water and feed were provided ad libitum. The housing environment was maintained at 60–65% humidity, and 25–28 °C temperature, to minimize the effects of environmental factors.

A total of 108 weaned piglets [Duroc × (Landrace × Yorkshire)], aged 28 days and weighing 7.72 ± 0.64 kg, were randomly assigned to three treatments, balanced by sex (half male/female). No antibiotics were used for experimental pigs before weaning or during the experiment. Each treatment consisted of six replicate pens with six piglets each. The experiment lasted 42 days and included two periods: (1) Copper-Rich period (CR, Days 0–28): a. the control treatment (CON) received a copper-free diet (no copper added); b. the inorganic copper (CS) treatment received a copper-free diet with 120 mg copper/kg from copper sulfate; c. the organic copper (CP) treatment received a copper-free diet with 120 mg copper/kg from copper-peptide (Alltech). (2) Copper-Free period (CF, Days 29–42): all treatments changed to a copper-free diet. The nutrient content of diets met or exceeded nutrient requirements NRC (2012)^5^. Feed samples were collected to detect the true copper contents. The formulation and nutrient composition of experimental diets and measured copper contents are listed in Supplementary Table 1. Body weights were measured on Days 0, 14, 28, and 42 of the experiment. Fresh fecal samples (*n* = 6 per treatment, middleweight piglets per pen were selected) were collected at Days 28 and 42 for metagenomic sequencing. Fecal sample information was as described in Supplementary Table 2. On Day 28, one pig per pen was fasted for 12 h in individual pens before being humanely euthanized via electrical stunning and exsanguination. Colon tissue and colonic digesta were subsequently collected for future detection. All samples were immediately frozen in liquid nitrogen and stored at −80 °C. Ten millimeter segments of proximal colon were flushed with PBS and then fixed in 4% paraformaldehyde for histology analysis. This study is reported in accordance with ARRlVE guidelines (https://arriveguidelines.org).

### Isolation and resistant phenotype testing of bacteria

To validate the antibiotic resistance phenotypes predicted by metagenomic analysis, bacterial isolates from fecal and colonic digesta samples were subjected to phenotypic resistance profiling. Briefly, the suspension of fresh feces and colonic digesta were gradient dilution, and cultured on MacConkey agar medium at 37 °C for 24 h. The selected bacterial colonies were further cultured on tryptic soy agar (TSA) medium for purification. 16S rRNA sequencing was performed to identify the isolates. Identified isolates were tested for antimicrobial susceptibility to 12 antibiotics using Kirby-Bauer disk diffusion, including Tetracycline (TE), erythromycin (ERY), gentamycin (CN), florfenicol (FFC), kanamycin (K), penicillin (PEN), ciprofloxacin (CIP), cefazolin (KZ), enrofloxacin (ENR), cefoxitin (FOX), streptomycin (S), and ampicillin (AMP). The antibiotic susceptibility was interpreted by Performance Standards for Antimicrobial Susceptibility Testing (CLSI, M100-S30). *E. coli* ATCC 25922 and *Enterococcus faecalis* ATCC 29212 served as quality control.

### Co-culture of epithelial monolayer and conjugative transfer system

To investigate the impact of copper sources, SCFAs, gut barrier integrity, and hypoxic conditions on the horizontal transfer of ARGs under copper exposure, a co-culture model combining an epithelial monolayer (constructed by cell line HT-29) and a conjugative transfer system [containing the donor (*E. coli* DH5α-plasmid RP4) and recipient (*E. coli* HB101)] was established using Transwell equipment. The conjugative transfer system was constructed according to the previous description^18^. Cell line HT-29 were cultured in DMEM medium supplemented with 10% (v/v) FBS (10099141, Gibco) and 10 mM HEPES buffer (15630106, Gibco), maintained at 37 °C with 5% CO_2_ and 95% air atmosphere with 90% humidity. No antibiotics were used in the medium. Cells were seeded on 6.5 mm Transwell polyester inserts (3 μm pore size, Costar, Corning Inc., Corning, NY, USA) at a density of 1 × 10^5^ cells per insert and cultured for 8~9 days to form a differentiated monolayer. For conjugative transfer, both donor and recipient strains were grown in LB medium at 37 °C, 150 rpm for 12~16 h, harvested by centrifugation (6000 × *g*, 5 min), washed three times with PBS (pH = 7.2), and resuspended to 50 mg/mL in PBS. Equal volumes of donor and recipient suspensions (1:1 ratio, 10^8^ cfu/mL each) were mixed and added to the apical chamber of the HT-29 monolayer for 12 h incubation. For the experimental treatment, we first treated the monolayer cell model and the conjugate transfer system with seven concentrations of copper (0, 0.5, 1.0, 5.0, 10.0, 50.0, and 100.0 μmol/L) to determine the optimal addition concentration of copper in the co-culture model (Supplementary Fig. 9). The treatment was as followed: a. Factor of copper sources: terile Milli-Q water (Con), 5 μM CuSO_4_ (CS), and 5 μM Cu-peptide (CP) were added to the co-culture model. b. Factor of butyrate: 5 μM Cu^2+^ (CS), and 1 mM butyrate + 5 μM Cu^2+^ (CS-Butyrate) were added to the co-culture model; Milli-Q water (Con), 5 μM CuSO_4_ (CS), 1 mM butyrate (Butyrate), and 1 mM butyrate + 5 μM Cu^2+^ (CS-Butyrate) were added to epithelial monolayers and conjugative transfer system, respectively. c. Factor of hypoxia: Two groups, 5 μM Cu^2+^ were added to the co-culture model. During the treatment period, the gas composition of the culture environment was altered. Hyperoxia: 5% CO_2_ and 95% air atmosphere; Hypoxia: 80% N_2_, 10% H_2_, and 10% CO_2_ (Sasaki et al.^18^). The co-culture model construction process does not involve hypoxic treatment. Transepithelial electrical resistance (TEER) was measured to assess the integrity of the epithelial monolayer. ROS generation and transconjugants were also detected as previously described in ref. ^19^.

### DNA extraction, library preparation, and shotgun metagenomic sequencing

Briefly, total genomic DNA was extracted from fecal sample of d 28 and d 42 pigs using the E.Z.N.A Soil DNA Kit (Omega Bio-tec, U.S.). Concentration and purity were determined with TBS-380 and NanoDrop2000 (Thermo Fisher Scientific, USA), respectively. The quality of extracted DNA was checked on 1% agarose gel. Then, DNA was fragmented to approximately 400 bp using Covaris M220 (Gene Company Limited, China). Adapters ligation, cleanup and enrichment were performed using NEXTFLEX Rapid DNA-Seq (Bioo Scientific, USA). Shotgun metagenomic sequencing was performed on Illumina NovaSeq/Hiseq Xten at Majorbio Bio-Pharm Technology Co., Ltd. (Shanghai, China).

### Quality control, host decontamination

Raw sequencing data was filtered to remove low-quality reads and adapter using fastp (version 0.19.4) with parameters “--cut_by_quality3 -W 4 -M 20 -n 5 -c -l 150 -w 3” ^20^. Filtered reads were then mapped to the pig genome to remove host contamination using bowtie2 (version 2.4.1)^21^. The resulting high-quality clean reads were used for downstream analysis.

### Metagenomics assembly, gene prediction and annotation

Megahit (version 1.2.9) was employed to assemble metagenomics reads with default parameters. Clean sequence reads of samples generated a set of contigs with “-min-contig-len 500” parameters^22^. Assembled scaffolds were processed to predict open reading frames (ORFs) using MetaProdigal (version 2.6.3)^23^. The predicted ORFs were clustered to establish a non-redundant (NR) gene catalog (95% identity over 90%) using CD-HIT (version 4.6.8) with option “-aS 0.9, -c 0.95” ^24^. The ARG, MRG, MGE, and taxonomy annotation were identified against the SARG database, BacMet database, MGE database, and NCBI-nr database, respectively, using blastp implemented in DIAMOND (version 2.0.9) with the following filter parameters (*e* value ≤ 1 × 10^−5^, coverage ≥ 40%, and identify ≥ 70%)^25^. The PlasFlow software was used to predict plasmid sequences for all ARG/MGR-carrying contigs^26^. The coverage of each contig was calculated by mapping clean reads to the contigs using bbmap (version 38.44) with the default parameters^27^. The abundance of a given type/subtype was calculated using the following formula^28^(1):

1 $${\mathrm{Abundance}}\left({\mathrm{coverage}},{\mathrm{t}}/{\mathrm{Gb}}\right)=\mathop{\sum }\limits_{1}^{n}\frac{N\times {L}_{\mathrm{reads}}/{L}_{\mathrm{NR}\,\mathrm{genes}}}{G}$$

where *n* represents the number of annotated gene type or subtypes, *N* represents the number of clean reads mapped to the NR gene, *L*_reads_ and *L*_NR reads_ represent the length of the clean reads and NR gene, and *G* represents the size of the metagenomic after filtering (Gb).

### Co-occurrence analysis and bacteria host of ARGs and MRGs

The bacterial hosts of ARGs and MRGs were identified by integrating metagenomic assembly with taxonomic annotation, as previous description^28–30^. The ORFs sequences on the contigs that carried ARGs- or MRGs-like ORFs were searched from NCBI RefSeq database using blastp with *e* value ≤ 10^−5^. The results were parsed by MEGAN (version 6), and the contigs were annotated as the same taxon (more than 50% of ORFs on the contigs). In addition, the previous pathogen list, including 1005 pathogenic species, was applied to identify pathogenic hosts in this study^31^. The co-occurrence patterns of ARGs/MRGs and MGEs were analyzed similarly to those of hosts. Briefly, MGEs including transposase, integrase, recombinase, and resolvase on ARGs- or MRGs-carrying contigs (ACCs, MCCs) were identified by aligning with the MGEs database to assess their transfer risk.

### Histological analysis

Morphological assessment and histological scoring were performed according to established criteria^14^. Briefly, colonic tissue sections (5 μm) were stained with hematoxylin and eosin (H&E). Following dehydration through absolute ethanol and xylene, slides were scanned using a VENTANA DP 200 slide scanner (Roche, USA). The digitized images were analyzed with Image Viewer v3.2 software to evaluate inflammatory infiltration, crypt architectural distortion, ulceration, crypt loss, and edema.

### Western blotting

Protein quantification was mainly used to assess colonic barrier integrity in piglets. Briefly, total colon protein was extracted with RIPA lysis buffer (Solarbio, Beijing, China) containing a protease inhibitor cocktail and quantified using a bicinchoninic acid (BCA) protein assay kit (Thermo Fisher Scientific, MA, USA). Proteins (300 ng per lane) were separated by SDS-PAGE and transferred to PVDF membranes. After blocking with 5% skimmed milk, the membranes were incubated overnight with primary antibodies against β-actin, MUC2, ZO-1, and E-cadherin (Abcam), followed by incubation with corresponding DyLight 800-labeled secondary antibodies (1:1000, Cell Signaling). Protein bands were visualized using an Odyssey Clx imaging system (4647 Superior Street, LI-COR Biotechnology, Lincoln, NE), and their densities were quantified with ImageJ v1.8.0 software after normalization to β-actin.

### Copper, SCFAs, and oxidative stress determination

Copper contents in feed, feces and colonic digesta were determined as our previous description^14^ using inductively coupled plasma-mass spectroscopy 7500 (ICP-MS, Agilent, USA).

SCFAs (i.e., acetate, propionate, butyrate) in feces and colonic digesta were quantified through the procedures of Wu et al.^32^, using the Ion chromatography (ICS-3000, Dionex, USA) with a Dionex IonpacTM AS11 analytical column (4 × 250 mm).

Reactive oxygen species (ROS) and Malondialdehyde (MDA) were used to assess the status of oxidative stress. ROS generation was measured using 2, 7-dichlorofluorescin diacetate (DCFH-DA) (BestBio, Shanghai, China) according to the instructions of the manufacturer. MDA was determined using an acid reactive substance (TBARS) assay, and its concentration was detected by HPLC. The specific method has been described in our previous study^33^.

### Statistical analysis

R software (version 3.6.3, https://www.r-project.org/) and GraphPad Prism (version 9.3.1) were used for the statistical analysis and plotting. Data are presented as means with standard deviation standard error of the mean (SEM). A *p*-value < 0.05 was considered statistically significant; a *p*-value between 0.05 and 0.10 was described as a non-significant trend and should be interpreted cautiously as exploratory observations. The experimental unit is the single animal. Variation partitioning analysis (VPA) based on partial redundancy analysis (RDA) was conducted using the vegan package, variables included fecal copper contents, abundance of MGE types, and microbial community structure. The rarefaction analysis based on Mothur (version 1.21.1) was conducted to reveal the diversity indices, including the richness and Shannon index. Principal coordinate analysis (PCoA) was performed to visualize the dissimilarity of ARG, MRG, and microbial composition in samples based on the Bray–Curtis distance metrics. Significant differences were analyzed employing the Anosim test. Linear regressions were based on the Pearson correlation coefficient. Differential taxa were identified by Linear discriminant analysis effect size (LEfSe). Throughout allocation, experimental conduct, outcome assessment, and data analysis, only Yang Wen and Wentao Lyu were aware of the group allocation.

## Results

### Differential responses of fecal resistome to (in-) organic copper in feed

**Fig. 1:**
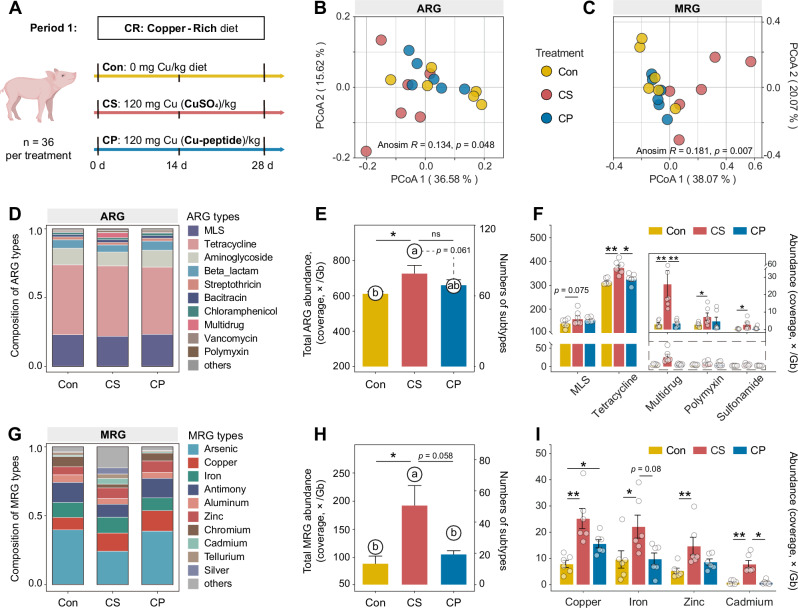
Comparison of fecal ARG and MRG profiles under dietary in- and organic copper. **A** Schematic of the weaned piglet experiment in period 1 (feeding copper-rich diet). **B, C** PCoA of ARG and MRG composition based on Bray-Curtis distance metrics (subtype level). Significant difference among the resistome structures was evaluated by Anosim test. **D, G** The proportions of top 10 ARG (MRG) types in the feces, other types are grouped into “others”. **E, H** Bars represent the total abundance of ARGs (MRGs) and circles represent the number of ARGs (MRGs), respectively. **F, I** Abundance of ARG (MRG) types that differed among groups. MLS, macrolide-lincosamide-streptogramin. CON, control group; CS, copper sulfate group; CP, copper-peptide group. The abundances of ARGs and MRGs are estimated as coverage normalized to data size (×/Gb). Values are shown as mean and error bars represent SEM. *n* = 6 piglets/groups. Statistical significance was analyzed with Kruskal–Wallis test. ns, non-significant; * represents significant differences in ARG (MRG) abundance; and different letters represent significant differences in ARG (MRG) numbers, *p* < 0.05.

To characterize the response of fecal resistome to inorganic copper (CS, CuSO_4_) and organic copper (CP, Cu-peptide) in feed (Fig. 1A), we employed metagenomic sequencing to profile the change patterns of ARGs and MRGs. Principal coordinate analysis (PCoA) revealed significant treatment effects on ARGs (ANOSIM *R* = 0.134, *p* = 0.048) and MRGs (ANOSIM *R* = 0.181, *p* = 0.007) (Fig. 1B, C). Notably, dietary CuSO_4_ induced broader disturbances in resistance gene structures compared with the CP group (Fig. 1D, G).

The total abundance and diversity of ARGs and MRGs were next quantified. Dietary CuSO_4_ increased the total abundance and diversity of ARGs compared to a copper-free diet (*p* < 0.05, Fig. 1E). In detail, multidrug was the most significant type that contributed to the increase of ARG diversity (Supplementary Fig. 1A, C); The resistant abundance of tetracycline, multidrug, polymyxin, and sulfonamide resistance significantly increased in the CS group (*p* < 0.05, Fig. 1F).

For MRGs, compared to both the CON group and CP group, the total abundance and diversity of MRGs significantly increased in the CS group (*p* < 0.05, Fig. 1H), mainly in copper, iron, zinc, and cadmium resistant types (*p* < 0.05, Fig. 1I). Both CuSO_4_ (*p* < 0.01) and Cu-peptide (*p* < 0.05) feed enhanced copper resistance. Dietary copper-peptide supplementation did not promote MRGs abundance and diversity as extensively as CuSO_4_, but mainly increased copper resistance (Supplementary Fig. 1B, D).

### Persistence of fecal resistome following dietary copper deprivation

**Fig. 2:**
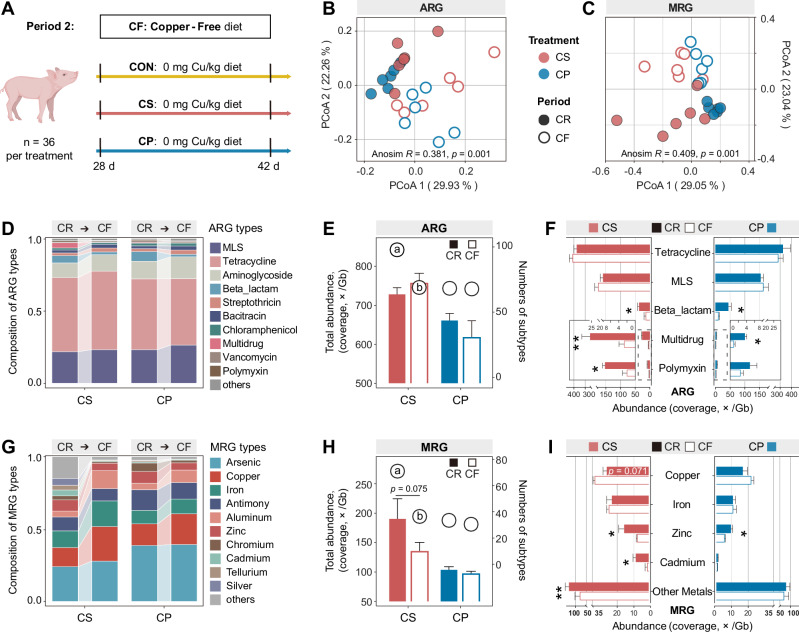
Persistence of fecal resistome following dietary copper deprivation. **A** Schematic of the weaned piglet experiment in period 2 (feeding copper-free diet). **B, C** PCoA of ARG and MRG composition based on Bray-Curtis distance metrics (subtype level). Significant difference among the resistome structures was evaluated by Anosim test. **D, G** The changing pattern of top 10 ARG (MRG) types proportions from copper-rich diet to copper-free diet. **E, H** Bars represent the total abundance of ARGs (MRGs) and circles represent the numbers of ARGs (MRGs), respectively. **F, I** Abundance of ARG (MRG) types that differed between CR and CF periods. MLS, macrolide-lincosamide-streptogramin. CS, copper sulfate group; CP, copper-peptide group; CR, copper-rich period; CF, copper-free period. Values are shown as mean and error bars represent SEM. *n* = 6 piglets/groups. Statistical significance was analyzed with Wilcoxon signed-rank test. ns, non-significant; * represents significant differences in ARG (MRG) abundance, *p < 0.05, **p < 0.01; and different letters represent significant differences in ARG (MRG) numbers, *p* < 0.05.

To evaluate the persistence of dietary copper effects on the fecal resistome, we conducted a copper deprivation experiment starting on Day 28, during which all groups were switched to a copper-free diet (Fig. 2A). PCoA showed significant shifts in both ARG (ANOSIM *R* = 0.381, *p* = 0.001) and MRG (ANOSIM *R* = 0.409, *p* = 0.001) profiles after copper deprivation (Fig. 2B, C). The CuSO₄ group exhibited the most distinct compositional changes in fecal ARG and MRG (Fig. 2D, G).

After CuSO₄ deprivation for 14 days, total abundance of ARGs remained elevated, though the diversity of ARGs declined (Fig. 2E). The increase in multidrug resistance induced by CuSO₄ rapidly diminished, while tetracycline and MLS resistance levels were largely stable (Fig. 2F). In the Cu–peptide group, ARG abundance and diversity had no significant difference. Following dietary CuSO₄ deprivation, the total abundance of MRG showed a non-significant decreasing trend (*p* = 0.075, non-significant), while the diversity of MRGs showed a significant reduction (*p* < 0.05). (Fig. 2H). The abundance of MRG types performed broadly decreased (except for copper and iron types) (*p* < 0.05, Fig. 2I and Supplementary Fig. 1B, D). Although copper resistance showed a trend toward enrichment (*p* = 0.071, non-significant), its magnitude of increase was markedly reduced compared to the copper-rich period. In contrast, dietary copper-peptide deprivation had minimal effects on MRG abundance and diversity.

### Potential factors shaping fecal resistome profiles

**Fig. 3:**
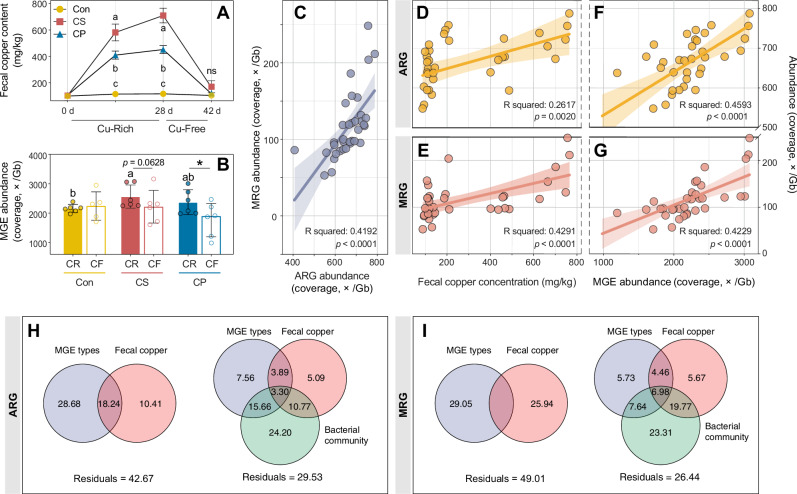
Driving factors influencing the development of ARG and MRG. **A** The changes of fecal copper concentration in feces. **B** The total abundance of MGE. **C** Pearson correlations between the total ARG abundance and total MRG abundance. **D**, **E** Pearson correlation between the fecal copper concentration and the total ARG (MRG) abundance. **F**, **G** Pearson correlation between the total MGE abundance and the total ARG (MRG) abundance. **H**, **I** Variation partitioning analysis for determining the contributions of different factors to the variations in ARG (MRG). CON control group, CS copper sulfate group, CP copper-peptide group, CR copper-rich period, CF copper-free period. Values are shown as mean and error bars represent SEM. *n* = 6 piglets/groups. Different letters represent significant differences; * represents significant differences; ns non-significant.

To identify key factors influencing fecal resistance, Pearson correlation and variation partitioning analyses (VPA) were performed. As expected, fecal copper levels increased significantly with copper supplementation and declined rapidly after withdrawal in both CuSO₄ and Cu–peptide groups, remaining higher in CuSO₄-treated piglets (*p* < 0.05, Fig. 3A). Fecal copper concentration was positively correlated with the total abundance of ARGs, MRGs, and MGEs (Fig. 3B, D-G). ARG and MRG abundances were also positively correlated (Fig. 3C), indicating potential similar drivers.

Then, we established an explanatory dataset including fecal copper concentration and MGE abundance (type level) to perform VPA analysis (Fig. 3H, I). Fecal copper and MGEs jointly explained much of the ARG variation, with 42.67% unexplained, and 49.01% of MRG variance remaining unexplained. When bacterial community composition was incorporated into the model, the explained variance increased by an additional 13.14% for ARGs and 22.57% for MRGs, highlighting microbial structure as an additional determinant. Body weight change in piglets showed no significant correlation with resistance gene abundance (Supplementary Fig. 2) and was therefore excluded from the VPA model.

### Mobility risk of ARGs and MRGs

**Fig. 4:**
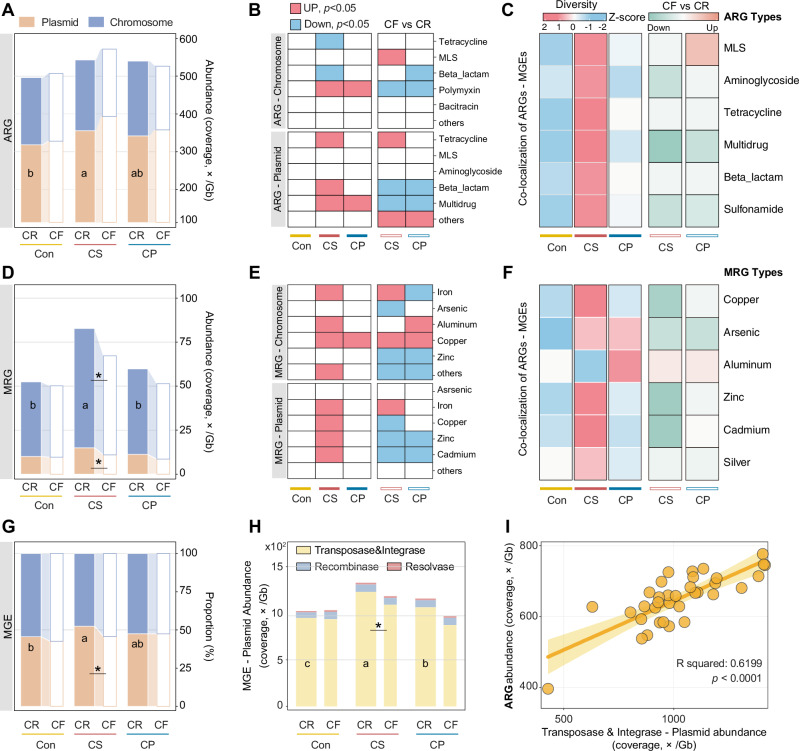
Co-localized patterns of ARGs, MRGs, or MGEs within the same contigs. Total abundance of ARG (**A**), MRG (**D**), or MGE (**G**) - carrying contigs annotated as plasmid and chromosome; Significant changes in the abundance of ARGs (**B**) and MRGs (**E**) types carried by plasmid, and chromosome. Numbers of ACCs (**C**) and MCCs (**F**) carrying MGEs. Values are normalized by Z-score. For the analysis between CF and CR periods, value means the number of CF minus CR, and normalized middle number with zero. **H** Abundance of transposase, integrase, recombinase, and resolvase–carrying contigs annotated as plasmid**. I** Pearson correlation between the ARG abundance and the sum abundance of transposase and integrase carried by the plasmid. *n* = 6 piglets/groups. Different letters represent significant differences, and * represents significant differences.

Plasmids and MGEs are key mediators of horizontal gene transfer. To assess their involvement, we analyzed the localization of ARGs and MRGs on plasmid- and chromosome-derived contigs. Although the overall proportion of plasmid and chromosomal contigs did not differ significantly among treatments (Supplementary Fig. 3), their propensities to carry resistance genes varied considerably (Fig. 4A, D). Dietary CuSO_4_ significantly increased the abundance of plasmid-carried ACCs and chromosomal MCCs (*p* < 0.05). Specifically, Plasmid-associated tetracycline, multidrug, β-lactamase, and metal (Fe, Cu, Zn, Cd) resistance genes were markedly enriched under CuSO₄ exposure, whereas chromosomal Fe, Al, and Cu resistance genes also increased (*p* < 0.05, Fig. 4B, E). Most of these enrichments declined after copper deprivation (*p* < 0.05), except for tetracycline, copper, and iron resistance.

We further examined the co-localized of MGEs and resistance genes within the same contigs. Dietary CuSO₄ also increased the diversity of ACCs and MCCs carrying MGEs (*p* < 0.05, Supplementary Fig. 4A, B). Consistent with the plasmid–chromosome distribution patterns, CuSO₄ significantly enhanced the diversity of tetracycline and multidrug resistance genes co-localized with MGEs, whereas the diversity of multidrug resistance types declined rapidly after copper deprivation (*p* < 0.05, Fig. 4C). For the diversity of MCCs-MGEs, copper, zinc, and cadmium resistance types increased in the CuSO₄ group but decreased sharply after copper deprivation (*p* < 0.05, Fig. 4F). Moreover, Dietary CuSO₄ increased the abundance of plasmid-carried transposase and integrase genes (Fig. 4G, H), which correlated positively with total ARG abundance (Fig. 4I). Contigs simultaneously carrying ARGs and MRGs were detected only under CuSO_4_ treatment (Supplementary Fig. 4C), mainly involving multidrug resistance genes co-localized with copper- and iron-resistance location. These findings suggest that CuSO₄ enhances ARG and MRG mobility through plasmid carrying and MGE co-localization, whereas MRG transmission appeared more dependent on chromosome-mediated genetic variation.

### Changes in fecal microbiome under dietary copper pressure

**Fig. 5:**
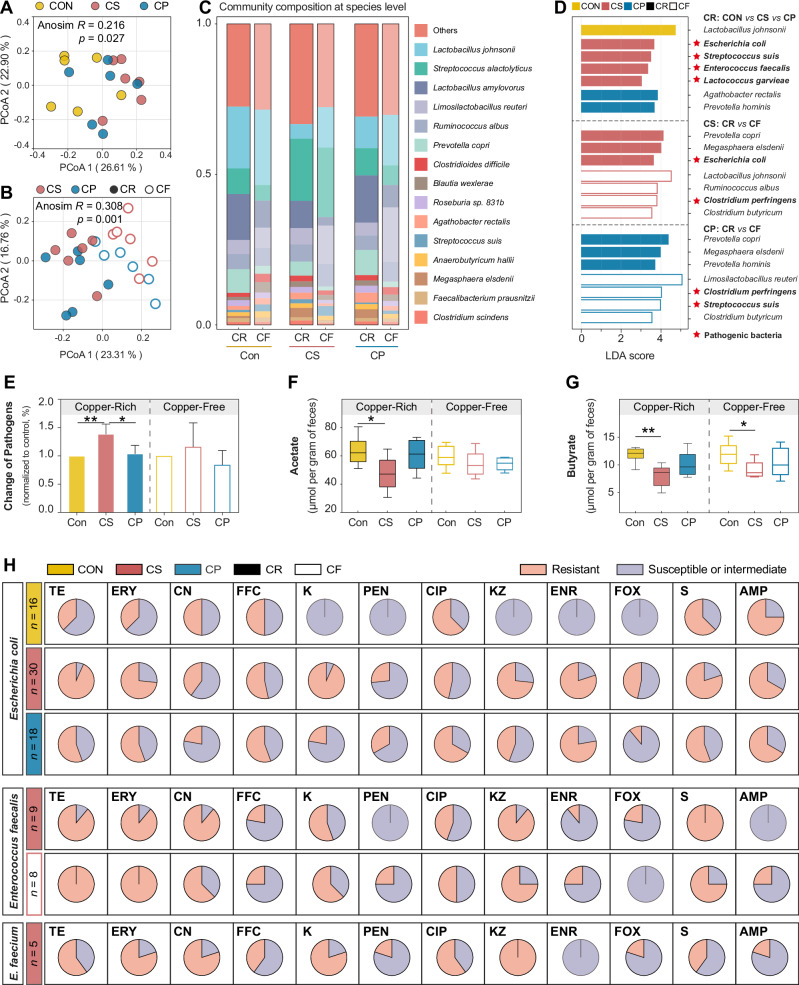
Dietary copper reshapes microbial community composition and raises resistant pathogenic bacteria abundance. **A, B** PCoA of community composition influenced by dietary copper and dietary copper deprivation based on Bray-Curtis distance metrics (species level). Significant difference among the community structures was evaluated by Anosim test. **C** The proportion of the top 10 abundant species, other species are grouped into “others”. **D** The most differential species are identified by LEfSe analysis, LDA threshold set as 3.0. Red star represents pathogenic bacteria. **E** Fold change of the pathogenic bacteria abundance. The concentration of acetate (**F**) and butyrate (**G**) in feces. **H** The antibiotic resistance profile of isolates from the feces and colonic digesta of piglets, specific data was shown in Figure S7. CON, control group; CS, copper sulfate group; CP, copper-peptide group; CR, copper-rich period; CF, copper-free period. Values are shown as mean and error bars represent SEM. *n* = 6 piglets/groups. *represents significant differences, *p < 0.05, **p < 0.01. Tetracycline (TE), aminoglycosides [gentamycin (CN), kanamycin (K), streptomycin (S)], beta-lactams [ampicillin (AMP), cefazolin (KZ), penicillin (PEN), cefoxitin (FOX)], quinolone [ciprofloxacin (CIP), enrofloxacin (ENR)], erythromycin (ERY), florfenicol (FFC), and cefazolin (KZ).

To investigate bacterial contributions to the fecal resistome, we examined the compositional changes of the fecal microbiota under copper exposure. Alpha diversity analysis showed that dietary CuSO₄ supplementation significantly increased the Shannon index at the species level (*p* < 0.05, Supplementary Fig. 5A, B), which returned to the control level after copper deprivation. PCoA showed differences in microbial community composition across treatments (Fig. 5A). After copper deprivation, community structures again shifted (Fig. 5B). During the copper-rich period, 7 differential species were identified (LDA > 3.0), including several potential pathogens enriched in the CuSO₄ group—*E. coli*, *Streptococcus suis*, *E. faecalis*, and *Lactococcus garvieae* (Fig. 5C–E). In contrast, the abundances of SCFAs–producing bacteria, such as *Lactobacillus johnsonii*, *Clostridium butyricum*, and *Lachnospiraceae NK4A136 group*, were significantly reduced (*p* < 0.05, Fig. 5C and Supplementary Fig. 5C, D). These taxa were partially enriched after CuSO_4_ deprivation (CS: CR vs. CF, Fig. 5D and Supplementary Fig. 5E). Similarly, the Cu–peptide group showed decreased abundances of several SCFA producers (*L. johnsonii*, *Limosilactobacillus reuteri*, and *C. butyricum*), but unlike CuSO₄, did not promote pathogen enrichment (CP: CR vs. CF, Fig. 5D and Supplementary Fig. 5F**)**. However, following Cu–peptide deprivation, pathogens including *Clostridium perfringens*, *S. suis*, and *Enterococcus faecium* became more abundant (CP: CR vs. CF, Fig. 5D and Supplementary Fig. 5F). Additionally, fecal concentrations of acetate and butyrate were significantly lower in the CuSO₄ group than in the control (*p* < 0.05, Fig. 5F, G), with butyrate levels remaining lower even after copper deprivation (*p* < 0.05).

### Dietary inorganic copper promotes resistant pathogenic bacteria development

To determine the bacterial hosts of resistance genes, we identified the top 20 ARG- and MRG-carrying species (Supplementary Fig. 6A, C). Dietary CuSO_4_ increased both the abundance and diversity of ARG-harboring pathogenic bacteria compared with the control, notably *S. suis* (MLS, aminoglycoside, beta-lactam)*, E. faecalis* (tetracycline, trimethoprim, aminoglycoside, MLS), and *E. coli* (multidrug, tetracycline, bacitracin, MLS). After copper deprivation, ARG abundance remained high in *E. faecalis* but declined sharply in *E. coli*. Pathogen–ARG carrying indicated that tetracycline, MLS, and multidrug resistance genes were predominant, with multidrug genes mainly carried by *E. coli* (Supplementary Fig. 6B). Different from ARGs, the top 20 MRG-carrying species were rare to carry multiple MRGs, while only in *Streptococcus alactolyticus* (Fe, Cu, Zn, Cd, Al), *E. coli* (Cu, Fe, Cd, Zn, As, Cr, Hg), and *S. suis* (Cu, Fe, Zn, Cd), and were more prevalent in the CuSO_4_ group (Supplementary Fig. 6C). In addition, dietary CuSO_4_ drove *E. faecalis* to become the dominant species that carried copper resistance genes (Supplementary Fig. 6D). As for dietary copper-peptide, it had minimal effects on host distribution of either ARGs or MRGs.

To further verify metagenomic predictions, we tested resistant phenotypes of isolates from feces and colonic digesta. A total of 64 *E. coli*, 22 *Enterococcus spp*. were identified (Fig. 5H and Supplementary Fig. 7). Dietary CuSO_4_ significantly increased *E. coli* resistance to tetracycline (28/30), beta-lactams [AMP (20/30), KZ (22/30), and FOX (14/30)], aminoglycosides [K (28/30), and S (26/30)], and ENR (24/30) compared to the Con group. *Enterococcus spp*. isolated from feces and colonic digesta in CS group were generally resistant to tetracycline (16/17), aminoglycosides [CN (13/17), S (15/17)], KZ (14/17), and ERY (16/17), but largely susceptible to beta-lactams [AMP (2/17), PEN (2/17), FOX (2/17)], and ENR (3/17).

### Colonic oxidative stress and barrier damage under copper exposure

**Fig. 6:**
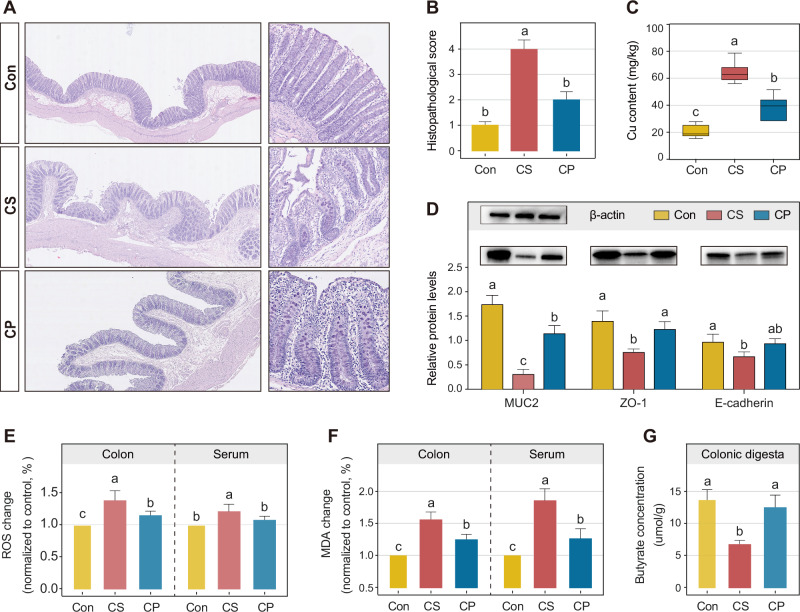
Responses of colonic barrier function, oxidative stress, and SCFAs to dietary copper supplementation. **A** H&E-stained colonic sections of three groups. **B** Histopathological scores of colons. *n* = 6 piglets/group. **C** Copper contents within colonic digesta. **D** Western blot of MUC2, ZO-1, and E-cadherin in the colon. E Fold change of the Fluorescence intensity relating to ROS levels (**E**) and MDA Production (**F**) within colon and serum. **G** Butyrate concentration in colonic digesta. CON, control group; CS, copper sulfate group; CP, copper-peptide group. Values are shown as means and error bars represent SEM. Statistical significance was determined using One-way ANOVA with Tukey’s test.

To investigate the mechanisms underlying copper-induced microbiota disturbance and resistance development, we observed the effects of dietary copper on gut homeostasis in piglets. Histological examination revealed more extensive bowel edema in the mucus layer, more loosely arranged intestinal glands, greater intestinal villus structure damage, and inflammatory cell infiltration in the CS group compared with Con and CP pigs, as confirmed by histological scoring (*p* < 0.05, Fig. 6A and B). Dietary Cu supplementation markedly increased copper accumulation in colonic digesta, with higher levels in the CS group than in the CP group (*p* < 0.05, Fig. 6C). Consistent with the histological findings, the expression of mucosal barrier proteins (MUC-2, ZO-1, and E-cadherin) was significantly reduced in the CS group (*p* < 0.05, Fig. 6D). Oxidative stress indicators, including ROS and MDA, were elevated in both colon tissue and serum of the CS group (*p* < 0.05, Fig. 6E, F), while colonic butyrate concentration was notably reduced (*p* < 0.05, Fig. 6G). Although Cu-peptide feeding also increased ROS production (*p* < 0.05, Supplementary Fig. 8), it did not significant difference in intestinal barrier proteins expression and butyrate concentration compared with the CON group.

### In vitro validation of intestinal homeostasis inhibits conjugative transfer of ARGs

**Fig. 7:**
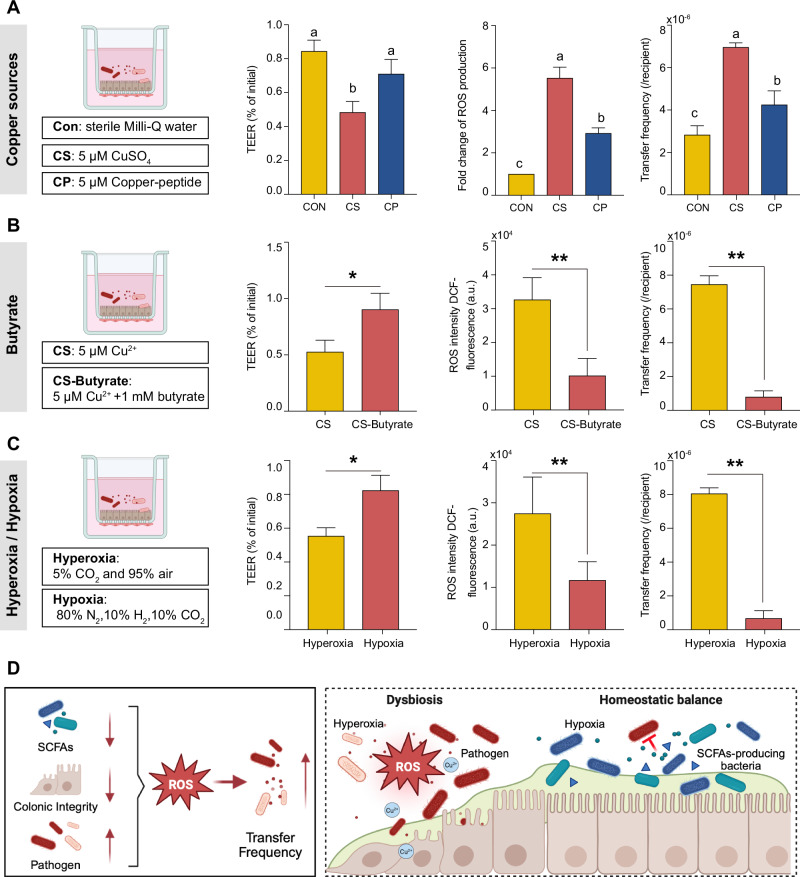
Effects of copper sources, butyrate, and state of hypoxia on the co-culture model (epithelial monolayer and conjugative transfer system) under copper exposure. Transepithelial electric resistance (TEER), Fluorescence intensity relating to ROS levels, and frequency of ARGs from the donor (*E. coli* DH5α) to the recipient (*E. coli* HB101) were detected to assess the epithelial barrier integrity, oxidative stress status, and conjugate transfer frequency. **A** For the factor of copper sources, sterile Milli-Q water (Con), 5 μM CuSO_4_ (CS), and 5 μM Cu-peptide (CP) were treated. **B** For the factor of butyrate, 5 μM Cu^2+^ (CS), and 1 mM butyrate + 5 μM Cu^2+^ (CS-Butyrate) were treated. **C** For the factor of hypoxia, Hyperoxia: 5% CO_2_ and 95% air atmosphere; Hypoxia: 80% N_2_, 10% H_2_, and 10% CO_2_. **D** Schematic of the association between gut dysbiosis and resistant transfer. Values are shown as means and error bars represent SEM, *n* = 3. Statistical significance was determined using a t-test (two treatments) and One-way ANOVA with Tukey’s test (three treatments).

To verify whether copper source, butyrate, and intestinal hypoxia influence the horizontal transfer of ARGs, we established a simplified co-culture model combining an epithelial monolayer and a conjugative transfer system using Transwell chambers. Copper exposure could induce a simultaneous increase in intestine-derived ROS production and decrease in TEER value, as well as an increase in bacteria-derived ROS production and conjugate transfer frequency (Supplementary Fig. 9). In the co-culture system, Cu-peptide exposure significantly mitigated Cu^2+^-induced epithelial barrier disruption, ROS accumulation, and transfer frequency (*p* < 0.05, Fig. 7A). Butyrate treatment increased TEER, and significantly decreased ROS and the frequency of conjugative transfer (Fig. 7B). The target of butyrate was further analyzed. Butyrate supplementation increased TEER and decreased intestine-derived ROS content primarily under Cu^2+^ exposure (*p* < 0.05), with minimal effects under normal conditions. In the single system of conjugative transfer, butyrate could reduce bacteria-derived ROS production, and conjugative transfer occurred in both the normal and Cu^2+^-exposed states (Supplementary Fig. 10). Given that copper-induced oxidative stress may disturb colonic hypoxia, we further simulated hypoxic conditions by adjusting the gas composition of the co-culture model. Compared with hyperoxia, hypoxia markedly increased TEER and significantly reduced ROS generation and conjugative transfer frequency (Fig. 7C).

## Discussion

This study found that dietary copper source critically shapes the development and persistence of the fecal resistome in pigs, primarily through modulating intestinal homeostasis. Dietary CuSO_4_ drove a broad and persistent expansion of ARGs and MRGs. The key mechanistic insight might be that dietary CuSO_4_-induced dysbiosis (characterized by oxidative stress, barrier dysfunction, and butyrate deficiency) facilitated the horizontal transfer of resistance genes, particularly those co-localized with MGEs on plasmids. In contrast, the stability of the gut environment under organic copper supplementation limited the mobility of resistance genes. These findings, validated by an in vitro co-culture model, shift the focus from metal selection alone to a more integrated pattern: the host’s intestinal health status acts as a central regulator, either permitting or restricting the spread of resistance genes within the gut microbiome under copper pressure.

Antimicrobial resistance genes transfer under heavy metal pressure has been widely reported in manure, soil, and water^34,35^. Copper ions in the environment generally create long-term selection pressure, enhancing antimicrobial resistance characterized by co-location with MGEs. However, the fecal resistome may behave differently, as dietary copper interacts with a complex intestinal environment over a shorter timescale. At a high CuSO₄ level (250 mg Cu/kg), dietary copper failed to co-select fecal ARGs or MGEs and instead reduced microbial abundance^36,37^, likely due to bactericidal effects that block horizontal transfer^10^. Considering that copper accumulates in feces, it still contributes to environmental resistance pollution. Interestingly, at a lower copper level (120 mg Cu/kg), dietary copper sulfate was more likely to promote the abundance and co-location of antimicrobial resistance genes and MGEs, similar to previous studies^38^. The increase of MGE was mainly classified as transposase and integrase, contributing to horizontal gene transfer among the community^39^. Notably, dietary copper sulfate (120 mg Cu/kg) maintained the richness and promoted the diversity of the community. It suggested that the intestinal community, when exposed to sublethal copper, might occur to non-lethal resistant mutations^40^, and tended to accumulate abundant antimicrobial resistance genes with increased potential dissemination. Additionally, the increase in ARG abundance, as measured by metagenomic sequencing, primarily reflects a shift in community composition and gene carriage under copper selection pressure. While this enrichment suggests that resistance traits confer a selective advantage in the presence of copper, our study does not directly measure these genes’ fitness in their bacterial hosts. The persistence of certain ARGs after copper withdrawal indicates that once established, resistant bacterial populations or associated genetic elements may maintain stability within the community; it also needs further validation through fitness assays.

The resistance development is strongly associated with MGE and intestinal community^41–43^. Under dietary copper sulfate selection stress, MLS, tetracycline, multidrug, polymyxin, and sulfonamide antibiotic-resistant types were similarly abundant in the intestinal contents and livestock environment^44,45^, and had a propensity to be carried by plasmid. It has been demonstrated that the frequency of plasmid-encoded antibiotic resistance genes transferred across bacterial genera by splicing was significantly enhanced^46,47^. Our study showed that inorganic copper exposure promoted the co-localization of ARGs with MGEs, especially transposases and integrases on plasmids. This pattern supports the hypothesis that ARG dissemination under copper pressure is mainly driven by horizontal gene transfer. Additionally, multiple ARGs were transmitted to pathogenic bacteria, mainly for *E. coli*, *Streptococcus*, and *Enterococcus*^48^. In addition, the experiment of dietary copper deprivation showed more information. As the selective pressure of copper decreased, probiotic and pathogenic bacteria re-entered competition for ecological niches, during which horizontal gene transfer may have continuously occurred. Potential probiotics gradually changed to the recipient bacterial populations of ARGs, such as *L. reuteri*^49^. However, only a few probiotic bacteria acquired antibiotic resistance genes. *L. johnsonii*, as the most significantly differentiated species, carried only a limited number of resistance genes. Interestingly, the *Clostridium spp*. enriched in the feces, which was generally identified as obligate anaerobes^50,51^. The appearance of *Clostridium* seemed to reduce the occurrence and dissemination of fecal ARGs in pigs^52^. These results further confirmed our hypothesis, suggesting that the community might be restructured after dietary copper deprivation, thus reducing the transmission of ARGs, though this needed to be validated by longer-term experiments.

As for metal resistance, an increase in abundance and diversity of most MRG types accompanying co-location with MGEs was observed, while this rapidly disappeared after dietary copper deprivation. Interestingly, MRGs were preferentially encoded by chromosomes, and this similarly disappeared after dietary copper deprivation. Genes encoding natural antimicrobial resistance were mainly located on the chromosomes of microbiota, and these resistance genes were vertically transferred to the next generation of bacteria^53^. Intestinal community could capture antimicrobial resistance genes from chromosomes and horizontally transfer them via transposase and integrase^54^. Considering that copper resistance remained highly abundant without carrying MGEs after dietary copper deprivation, the mobility manner of MRGs might be different. We hypothesized that dietary copper sulfate tended to accumulate MRGs primarily rely on chromosomal mutation or vertical inheritance. When the MCCs were carrying with MGEs, their host would share genetic information to accumulate abundant MRGs, although these changes were not persistent after dietary copper deprivation. For instance, iron-resistant genes without carrying MGEs were mainly found in *S. alactolyticus*, and their abundance was closely linked to the abundance of this species^55^. Copper-resistant genes co-located with MGEs were found in diverse species, with *E. faecalis* being the primary carrier^13^. Consistent with ARGs, the abundance of *E. faecalis* remained high and carried abundant copper resistance genes; *Escherichia coli* was identified as the main carrier strain of horizontally transferred MRGs. Additionally, it has been widely reported that copper could induce co-occurrence of ARG and MRG in microbial community^28,34^. Although our study also found that ARGs and MRGs were frequently co-localized within the same contigs, indicating potential genetic proximity or shared microbial hosts under copper exposure. However, such co-localization does not necessarily imply physical linkage or functional co-selection, which warrants further experimental validation.

In vitro, the prevailing mechanism by which sublethal copper ions exposure facilitated the horizontal transfer of antimicrobial resistance genes across bacteria has been generally profiled: a, sublethal copper ions exposure increased ROS production; b, bacterial SOS response pathways (triggered by DNA damage from reactive oxygen species) upregulate the expression of integrases and transposases, thereby enhancing the excision, transfer, and integration of MGEs carrying ARGs and MRGs^10,46,56^. In vivo, the change of ROS generation in the colon and serum indicated that the intestine and its community were in a state of oxidative stress under copper sulfate exposure. However, it might be difficult for copper ions to catalyze ROS production in the post-intestinal tract due to the hypoxic environment. Similar to a previous study^14^, dietary copper sulfate restrained the enrichment of short-chain fatty acid-producing bacteria (especially for butyrate) and reduced beta-oxidation activity, leading to a shift from an anaerobic to an aerobic environment in the colon, which increased (facultative) aerobic pathogens. Notably, butyrate is essential for maintaining intestinal integrity and anaerobic homeostasis^57^. At the host level, excessive Cu^2+^ could cause oxidative stress and barrier damage in intestinal epithelium, leading to increased ROS production, which aggravates the conjugate transfer of resistance genes^24^. Integrating these observations, we propose a hypothesis of copper-driven resistance propagation: a. inorganic copper-induced oxidative stress and the loss of SCFA-producing bacteria disrupt intestinal barrier function and oxygen gradients, thereby creating a favorable niche for facultative and aerobic pathogens that serve as ARG reservoirs and recipients; b. ROS triggered bacterial SOS response pathways to promote ARG transfer through enhancing co-localization with plasmid-carried MGEs. In vitro co-culture experiments further confirmed that butyrate supplementation restored epithelial resistance to oxidative stress and suppressed conjugative transfer frequency, while hypoxic conditions also reduced transfer events, highlighting the importance of intestinal homeostasis in modulating resistance mobility. Our proposed mechanisms are summarized in Fig. 7D.

In the present study, organic copper is clearly a better dietary copper option, which appeared to retain its growth-promoting effects while significantly reducing the accumulation of copper in feces and the dissemination of antimicrobial resistance genes. Due to its high bioavailability in the small intestine, the microbial community in the hindgut segment is under less copper selective pressure^16^. Notably, dietary copper-peptide supplementation still led to an increase in copper resistance genes, a predictable adaptive response to copper bioavailability. These were not linked to mobile genetic elements or antibiotic resistance, indicating a confined and lower-risk selection. Furthermore, the persistent enrichment of any resistance determinant entails a potential evolutionary risk. Additionally, hydrolyzed soy protein as its ligand contributed to the development of the intestinal community^58^. Previous studies found that dietary amino acid-chelated copper significantly increased probiotic abundance and decreased pathogenic bacterial abundance compared to the CuSO_4_ group in pigs^59^, similar to the current results. However, the increase in some opportunistic pathogens following copper-peptide deprivation suggests that its ecological impact is not entirely risk-free. This observation highlighted that any shift in trace mineral form or concentration might induce dysbiosis, though a far lesser degree than CuSO_4_. Therefore, organic copper should be viewed as a substantially safer alternative within animal production.

This is the first study to profile the transfer and persistence of fecal resistome with dietary (in-) organic copper in piglets, highlighting the potential compound and persistent pollution of fecal ARGs and MRGs induced by dietary inorganic copper. In addition, this study suggests that intestinal homeostasis acts as a key regulator of antimicrobial resistance transmission, specifically, dietary inorganic copper induced microbial disturbance, which may lead to an oxidative stress environment that facilitates the mobility of resistance genes among pathogens. These insights carry direct implications for designing nutritional strategies to mitigate antimicrobial resistance risks in animal production systems. The clear contrast between copper sources highlighted that replacing inorganic copper with organic alternatives (e.g., copper-peptide) represents a practical, sustainable strategy to maintain growth performance while substantially reducing the selection and dissemination of resistance genes in the gut and their subsequent environmental transfer.

Furthermore, a considerable fraction of variance remained unexplained in the VPA analysis. This unexplained component likely reflects the inherent complexity of the gut ecosystem and the influence of additional factors not explicitly captured in the current model. These may include host immune factors, phage-mediated gene transfer, stochastic community assembly, or unmeasured environmental covariates. Future work incorporating host transcriptomics, virome profiling, and longitudinal sampling could help disentangle these additional contributors to resistome transfer.

## Supplementary information

Supplementary information

## Data Availability

The characteristics and sources of the used data set are shown in the Supporting Information. Metagenomic sequencing raw data are available in the NCBI Sequence Read Archive (SRA) repository under accession number PRJNA1363772.
