# Supplementary material for: Dietary copper-driven colonic dysbiosis mediates oxidative stress and butyrate deficiency to facilitate the spread of resistome in pigs
Source: NPJ Biofilms Microbiomes. 2026 Mar 5;12:80. doi: 10.1038/s41522-026-00949-1 (PMC13084004; doi:10.1038/s41522-026-00949-1)
Supplement: Supplementary file 1 — Supplementary information [file 41522_2026_949_MOESM1_ESM.pdf]

Supplementary Information

Dietary copper-driven colonic dysbiosis mediates oxidative stress and butyrate deficiency to facilitate the spread of resistome in pigs

Yang Wen[a], Meng Gao[c], Zhenyu Wang[b], Xiaoyi Liu[b], Yunhui Zhang[d], Gang Lin[e], Pingli He[b], Hua Yang[a]*, Yingping Xiao[a]*, Wentao Lyu[a]*

Correspondence

Wentao Lyu (lvwt@zaas.ac.cn), Yingping Xiao (xiaoyp@zaas.ac.cn), and Hua Yang (yanghua@zaas.ac.cn). State Key Laboratory for the Quality and Safety of Agro-Products, Zhejiang Provincial Key Laboratory of Agricultural Microbiomics, Institute of Agro-Product Safety and Nutrition, Zhejiang Academy of Agricultural Sciences, Hangzhou, 310021, China

Supplementary Table 1. Formulation and nutrient composition of experimental diets (%, as-fed basis)

Ingredients	%
Corn	60.79
Soybean meal	18.00
Soy protein concentrate	2.00
Soybean oil	2.07
Extruded soybean	8.00
Fish meal, 64.6%	2.00
Protein whey powder, 3.8%	4.00
L-Lysine	0.40
DL-Methionine	0.09
L-Tryptophan	0.02
L-Threonine	0.13
Limestone	0.90
Dicalcium Phosphate	0.80
NaCl	0.30
Premix[a]	0.50
Total	100.00
Nutrient composition (%)	
DE (kcal/kg)	3499
CP	18.98
Dig Lys	1.23
Dig Met	0.36
Dig Trp	0.20
Dig Thr	0.74
Ca	0.70
P	0.54

[a] Premix provided the following per kilogram of feed: vitamin A, 12,000 IU; vitamin D, 2,500 IU; vitamin E, 30 IU; vitamin K, 3.0 mg; vitamin B12, 12 μg; D-pantothenic acid, 10 mg; nicotinic acid, 30 mg; choline chloride, 400 mg; Mn, 40 mg; Zn, 100 mg; Fe, 90 mg; I, 0.35 mg; Se, 0.3 mg. The added copper contents were calculated based on copper element, CON group: added 0 mg/kg, measured copper content 4.26 mg/kg; CS group: added 120 mg (Cu) /kg from $CuSO_4$, measured copper content 121.32 mg/kg; CP group: added 120 mg (Cu) /kg from Cu-peptide, measured copper content 117.38 mg/kg.

Supplementary Table 2. Sample information of experiments.

**Period1: Copper-Rich treatment**				
SampleName	Treatment	Segment	Sequencing	Animal
pig_feces_CON1	CON	Feces	Metagenomics	pig
pig_feces_CON2	CON	Feces	Metagenomics	pig
pig_feces_CON3	CON	Feces	Metagenomics	pig
pig_feces_CON4	CON	Feces	Metagenomics	pig
pig_feces_CON5	CON	Feces	Metagenomics	pig
pig_feces_CON6	CON	Feces	Metagenomics	pig
pig_feces_CS1	CS	Feces	Metagenomics	pig
pig_feces_CS2	CS	Feces	Metagenomics	pig
pig_feces_CS3	CS	Feces	Metagenomics	pig
pig_feces_CS4	CS	Feces	Metagenomics	pig
pig_feces_CS5	CS	Feces	Metagenomics	pig
pig_feces_CS6	CS	Feces	Metagenomics	pig
pig_feces_CP1	CP	Feces	Metagenomics	pig
pig_feces_CP2	CP	Feces	Metagenomics	pig
pig_feces_CP3	CP	Feces	Metagenomics	pig
pig_feces_CP4	CP	Feces	Metagenomics	pig
pig_feces_CP5	CP	Feces	Metagenomics	pig
pig_feces_CP6	CP	Feces	Metagenomics	pig
**Period2: Copper-Free treatment**				
pig_feces_CONS1	CON	Feces	Metagenomics	pig
pig_feces_CONS2	CON	Feces	Metagenomics	pig
pig_feces_CONS3	CON	Feces	Metagenomics	pig
pig_feces_CONS4	CON	Feces	Metagenomics	pig
pig_feces_CONS5	CON	Feces	Metagenomics	pig
pig_feces_CONS6	CON	Feces	Metagenomics	pig
pig_feces_CSS1	CS	Feces	Metagenomics	pig
pig_feces_CSS2	CS	Feces	Metagenomics	pig
pig_feces_CSS3	CS	Feces	Metagenomics	pig
pig_feces_CSS4	CS	Feces	Metagenomics	pig
pig_feces_CSS5	CS	Feces	Metagenomics	pig
pig_feces_CSS6	CS	Feces	Metagenomics	pig
pig_feces_CPS1	CP	Feces	Metagenomics	pig
pig_feces_CPS2	CP	Feces	Metagenomics	pig
pig_feces_CPS3	CP	Feces	Metagenomics	pig
pig_feces_CPS4	CP	Feces	Metagenomics	pig
pig_feces_CPS5	CP	Feces	Metagenomics	pig
pig_feces_CPS6	CP	Feces	Metagenomics	pig

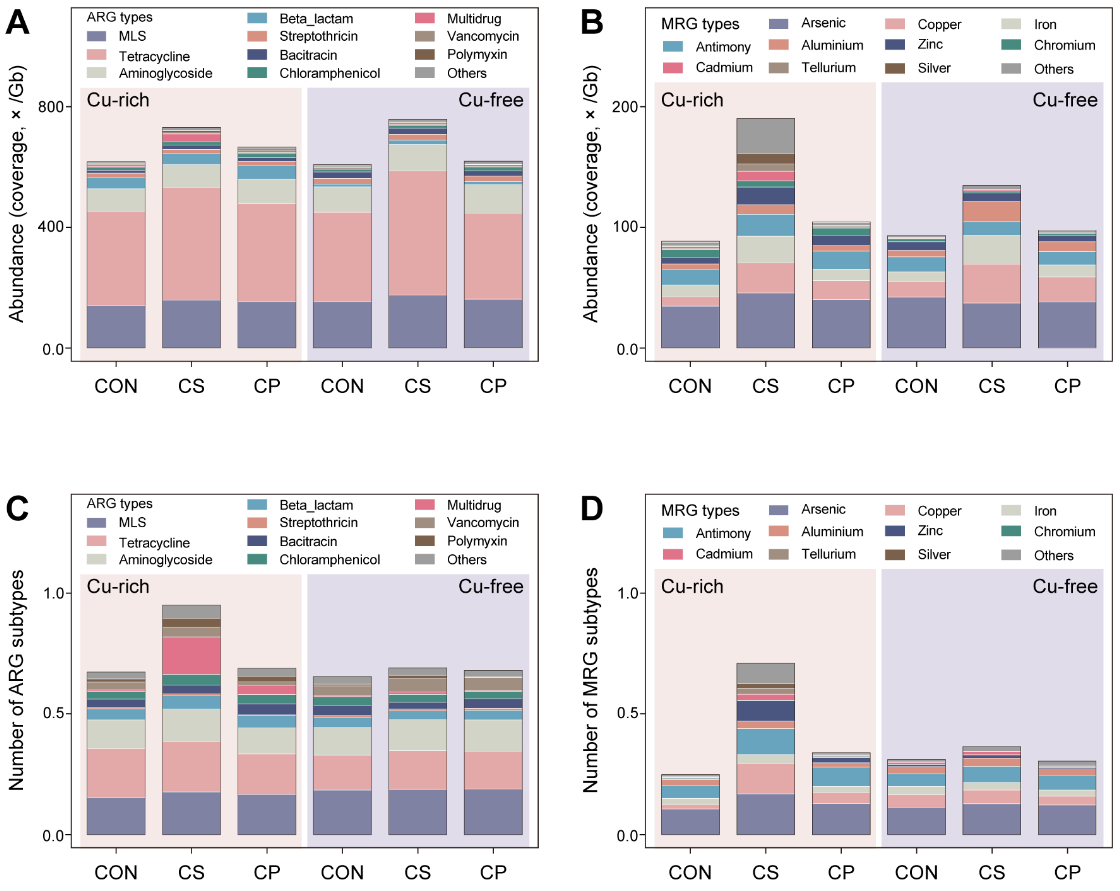

Supplementary Fig. 1. Abundance and numbers of ARG and MRG during the experiment, related to Figure 1 and Figure 2. A The abundance of top 10 abundant ARG types. **B** The abundance of top 10 abundant MRG types. **C** The number of top 10 abundant ARG types. **D** The number of top 10 abundant MRG types. The rest of ARG or MRG types are grouped into “Others”. CON, control group; CS, copper sulfate group; CP, copper-peptide group; CR, copper-rich period; CF, copper-free period. The abundances of ARGs and MRGs are estimated as coverage normalized to data size (×/Gb). *n* = 6 piglets/groups.

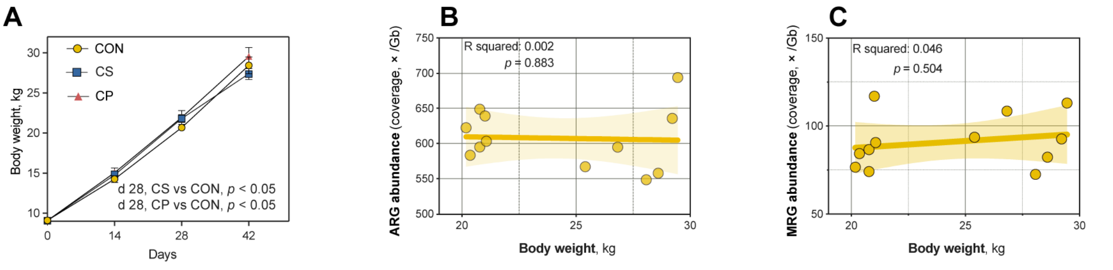

Supplementary Fig. 2. The body weight change in the experiment and its association with total ARG/MRG abundance, related to Figure 3. A Body weight change in piglets fed different diets. n = 36 piglets/groups. **B-C** Pearson correlation between the body weight and the total ARG (MRG) abundance. n = 6 piglets/groups. CON, control group; CS, copper sulfate group; CP, copper-peptide group. The abundances of ARGs and MRGs are estimated as coverage normalized to data size (×/Gb).

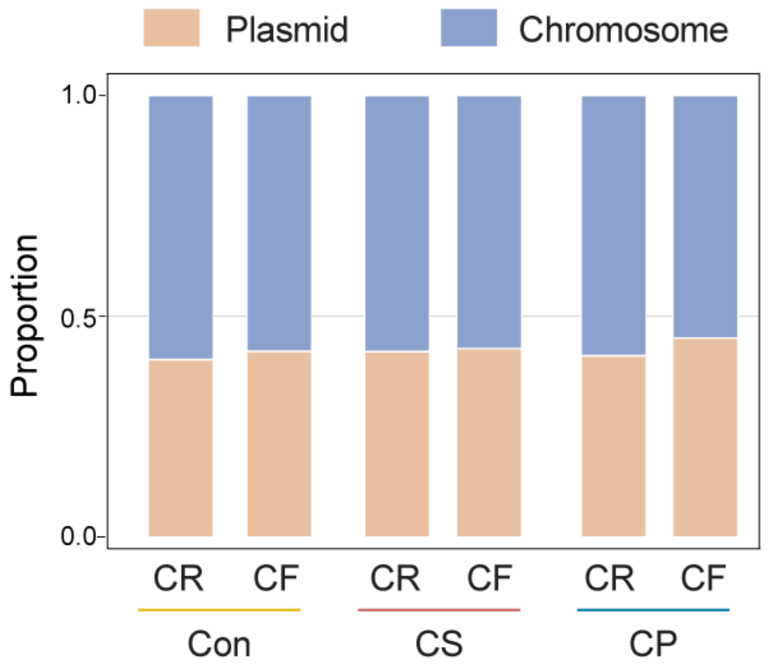

Supplementary Fig. 3. Proportion of contigs encoded by plasmids and chromosomes, related to Figure 4. CON, control group; CS, copper sulfate group; CP, copper-peptide group; CR, copper-rich period; CF, copper-free period.

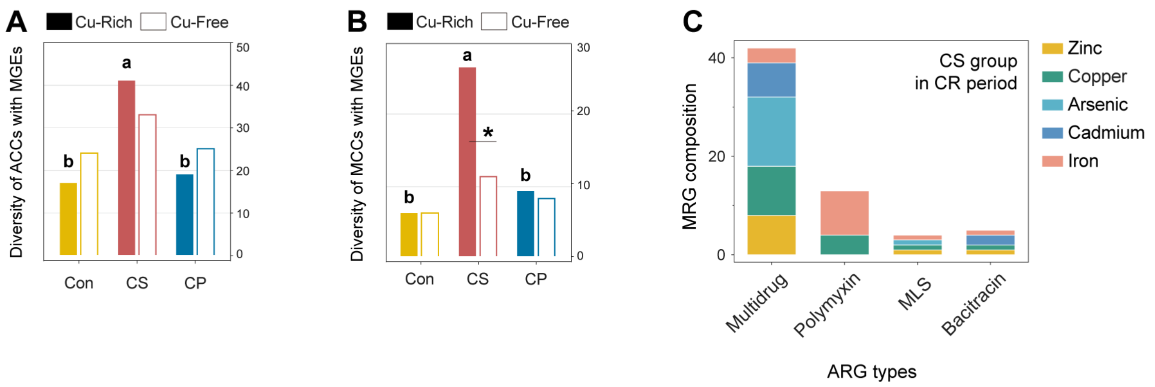

Supplementary Fig. 4. Co-occurrence patterns of resistance genes on one contig, related to Figure 4. A, B Numbers of ACCs (MCCs) carrying MGEs. **C** Compositions of MRG types co-located with each ARG type. CON, control group; CS, copper sulfate group; CP, copper-peptide group; CR, copper-rich period; CF, copper-free period.

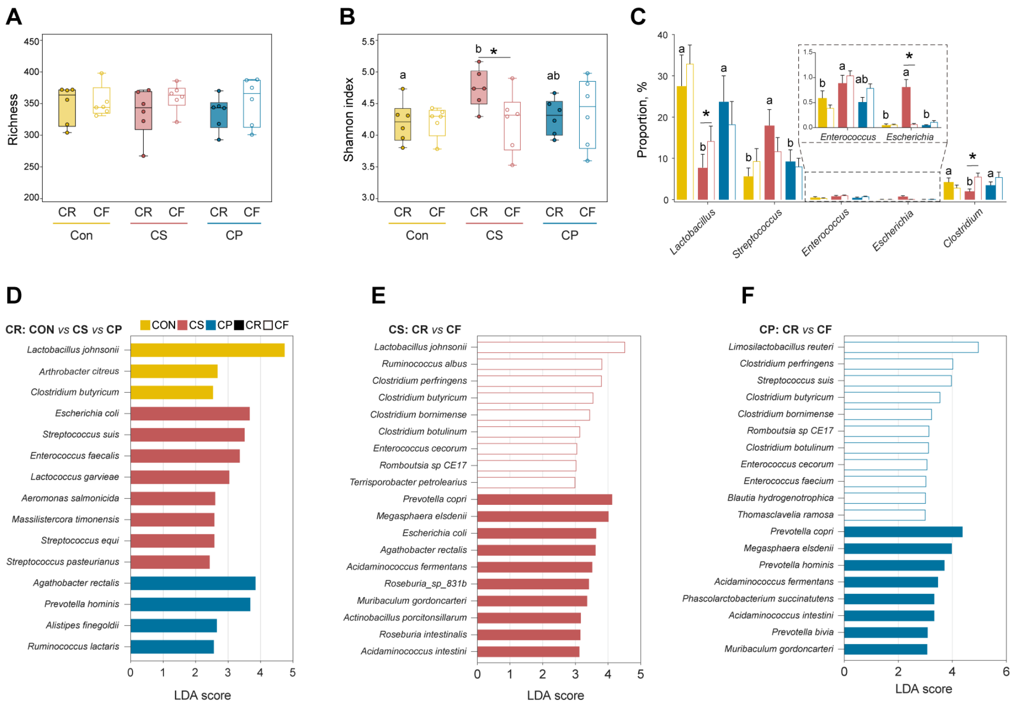

Supplementary Fig. 5. Alpha diversity and LEfSe analysis of fecal community, related to Figure 5. A Richness of fecal community at species level. **B** Shannon index of fecal community at species level. **C** The proportion of the key genera. The most differential species are identified by LEfSe analysis: **D** CON *versus* CS *versus* CP groups on the copper-rich period, LDA threshold set as 2.0; **E** CR versus CF in CS group, LDA threshold set as 3.0; **F** CR versus CF in CP group, LDA threshold set as 3.0. CON, control group; CS, copper sulfate group; CP, copper-peptide group; CR, copper-rich period; CF, copper-free period. n = 6 piglets/groups. Comparison among CON, CS, and CP groups was analyzed with Kruskal-Wallis test, and different letters represent significant differences; Comparison between CR and CF periods was analyzed with Wilcoxon signed-rank test, and * represents significant differences; ns, non-significant.

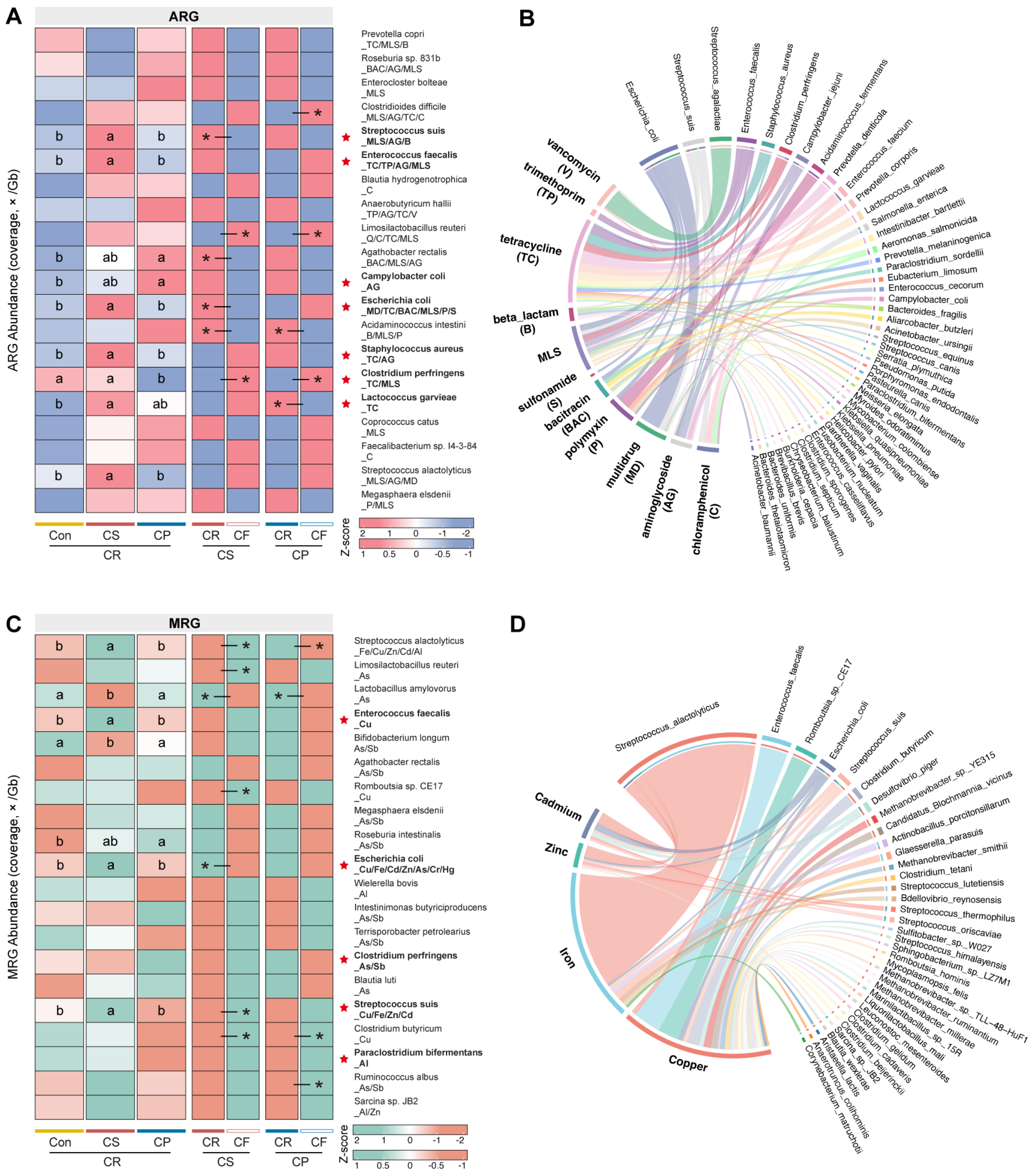

Supplementary Fig. 6. Hosts of ARGs and MRGs in dietary copper-influenced feces. A, C Top 20 abundant ARG (MRG)-carrying species in feces. The types of ARG (MRG) carried by species are listed. ARG (MRG)-carrying species are ordered from high to low in sum ARG (MRG) abundance. Values are normalized by Z-score. Red star represents pathogenic bacteria. **B** The number of contigs carried ARGs hosted by pathogens. **D** The number of contigs carried primarily differential metal resistance genes (including copper, iron, zinc, and cadmium) hosted by species. CON, control group; CS, copper sulfate group; CP, copper-peptide group; CR, copper-rich period; CF, copper-free period. The abundances of ARGs and MRGs carried by species are

estimated as coverage normalized to data size (×/Gb). $n = 6$ piglets/groups. Comparison among CON, CS, and CP groups was analyzed with Kruskal-Wallis test, and different letters represent significant differences; Comparison between CR and CF periods was analyzed with Wilcoxon signed-rank test, and * represents significant differences; ns, non-significant.

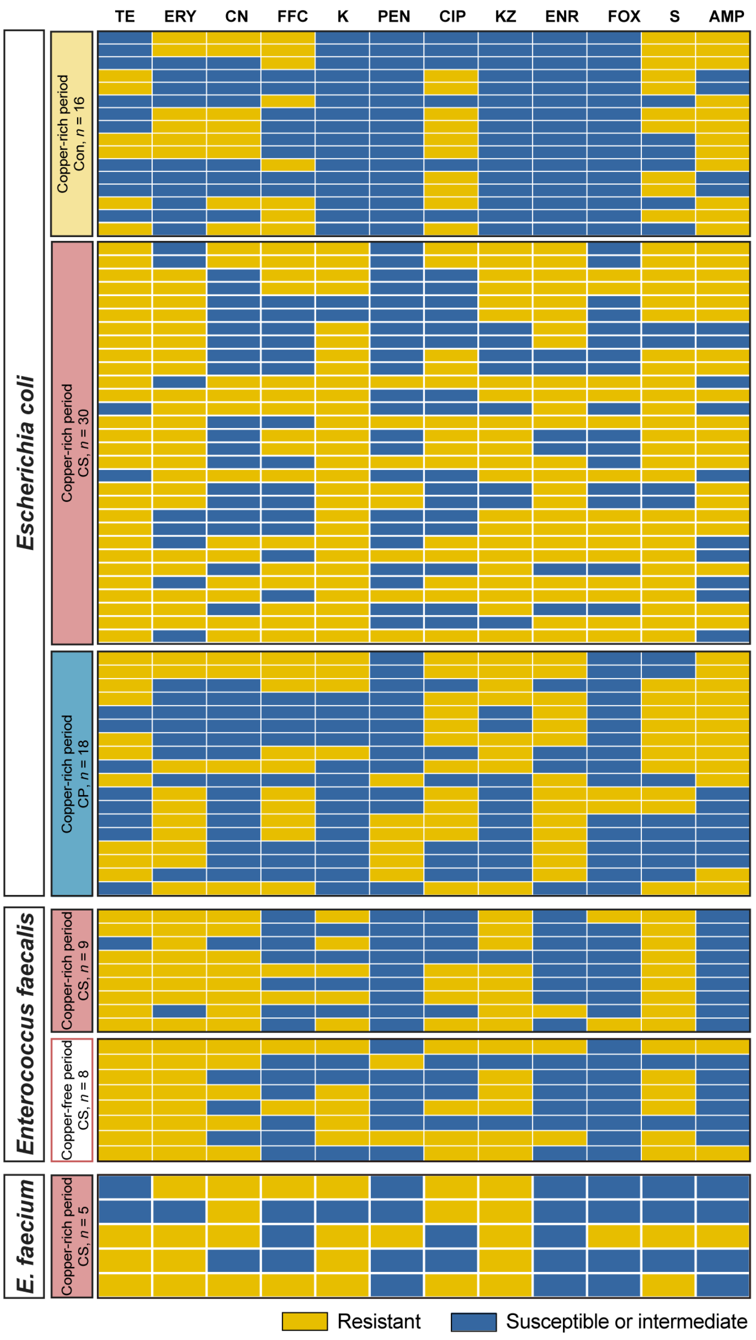

Supplementary Fig. 7. The antibiotic resistance profile of isolates (*Escherichia coli* and *Enterococcus spp.*) from feces and colonic digesta of piglets. CON, control group; CS, copper sulfate group; CP, copper-peptide group. tetracycline (TE), erythromycin (ERY), gentamycin (CN), florfenicol (FFC), kanamycin (K), penicillin (PEN), ciprofloxacin (CIP), cefazolin (KZ), enrofloxacin (ENR), cefoxitin (FOX), streptomycin (S), and ampicillin (AMP).

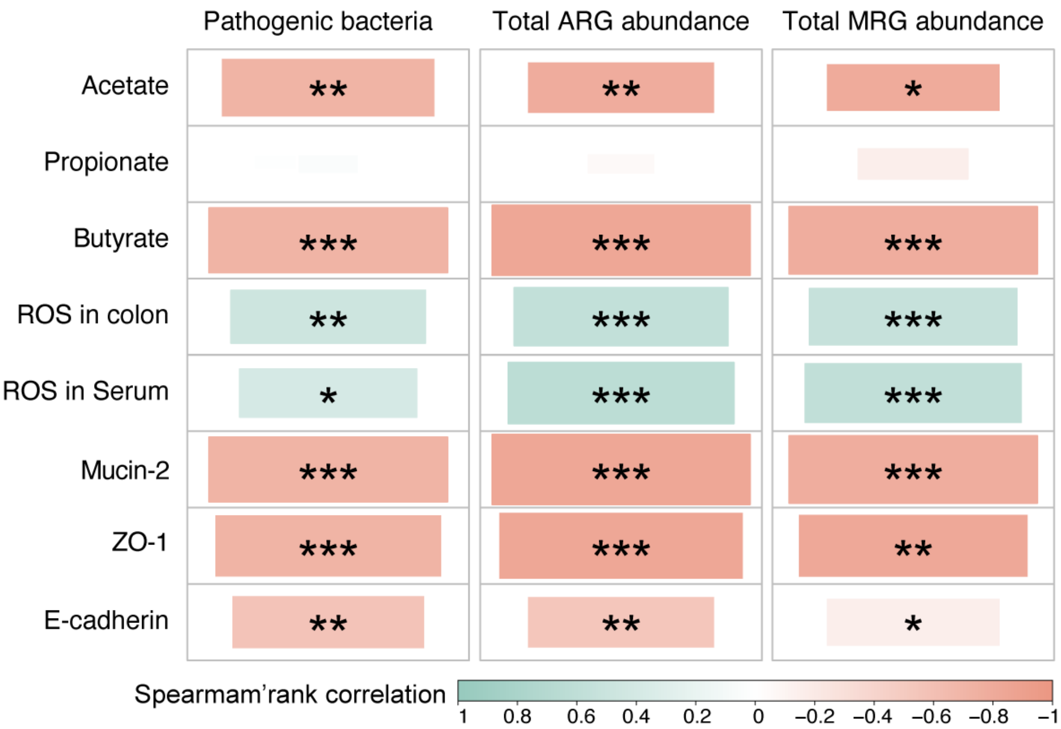

Supplementary Fig. 8. Spearman association among SCFAs concentration, oxidative stress, colonic barrier, pathogen abundance, ARG abundance, and MRG abundance.

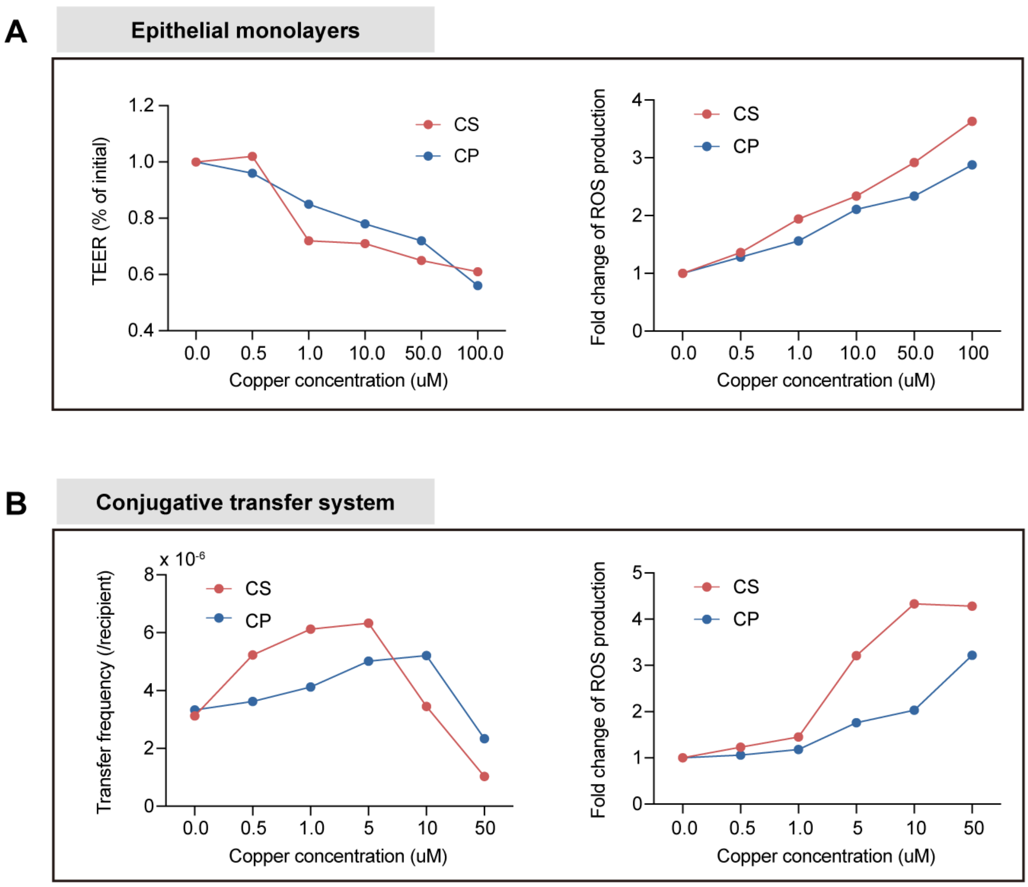

Supplementary Fig. 9. Effects of varying exposure concentrations of copper sulfate and copper peptide on the epithelial monolayer and conjugative transfer system. A For the epithelial monolayer model, Transepithelial electric resistance (TEER) and Fluorescence intensity relating to ROS levels were detected under Cu^{2+} and Cu-peptide exposure for 12h. **B** For the Conjugative transfer system, frequency of ARGs from the donor (*E. coli* DH5α) to the recipient (*E. coli* HB101) and ROS levels were detected under Cu^{2+} and Cu-peptide exposure for 12h. ROS level was normalized to the initial level. CS, copper sulfate group; CP, copper-peptide group.

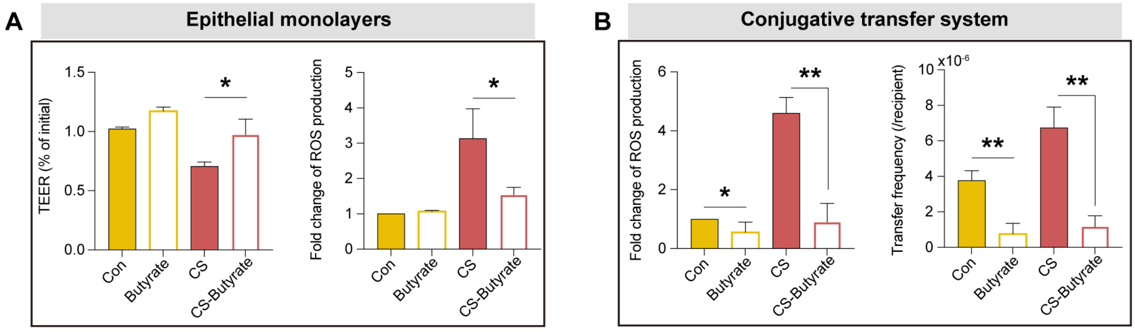

Supplementary Fig. 10. Effects of butyrate on the epithelial monolayer and conjugative transfer system under Cu^{2+} exposure, respectively. **A** For the epithelial monolayer model, TEER and Fluorescence intensity relating to ROS levels were detected under Cu^{2+} exposure for 12h. **B** For the Conjugative transfer system, frequency of ARGs from the donor (*E. coli* DH5α) to the recipient (*E. coli* HB101) and ROS levels were detected under Cu^{2+} exposure for 12h. ROS level was normalized to the Con group. Con, sterile Milli-Q water; Butyrate, 1 mM butyrate; CS, 5 μM Cu^{2+} exposure; CS-Butyrate, 1 mM butyrate + 5 μM Cu^{2+}. Comparison between Con and Butyrate groups (CS and CS + butyrate groups) was analyzed with t-test, and * represents significant differences.
